# Pvalb8, a Type of Oncomodulin, Regulates Neuromast Development and Auditory Function in Zebrafish

**DOI:** 10.3390/cells14191572

**Published:** 2025-10-09

**Authors:** Guiyi Zhang, Qianqian Li, Ying Xu, Hanmeng Zhao, Chao Yang, Dong Liu, Jie Gong

**Affiliations:** 1 School of Life Sciences, Nantong University, Nantong 226001, China; 2309310004@stmail.ntu.edu.cn (G.Z.); lqianqian2022@126.com (Q.L.); 2409310002@stmail.ntu.edu.cn (Y.X.); 2409110106@stmail.ntu.edu.cn (H.Z.); 2 Key Laboratory of Neuroregeneration of Jiangsu and Ministry of Education, Co-Innovation Center of Neuroregeneration, Nantong University, Nantong 226001, China; 2225510003@stmail.ntu.edu.cn

**Keywords:** hearing loss, calcium-binding protein, *pvalb8*, hair cells, zebrafish

## Abstract

**Highlights:**

**What are the main findings?**

- *pvalb8* is highly and specifically expressed in supporting cells and hair cells.
- The *pvalb8* mutation causes developmental defects in neuromasts and significant hearing loss.
- Loss of *pvalb8* suppresses supporting cell proliferation, limiting hair cell differentia-tion.
- Activation of the Wnt signaling pathway rescues the hair cell loss observed in *pvalb8* mutants.

**What is the implication of the main finding?**

- *pvalb8* regulates the development of neuromast and hair cells by mediating the Wnt signaling pathway.

**Abstract:**

Congenital hearing loss, frequently resulting from defective hair cells, remains poorly understood due to the incomplete identification of key pathogenic genes. Oncomodulin (OCM) is a kind of calcium-binding protein (CaBP) that regulates diverse cellular processes and is thought to play crucial roles in auditory function. In teleost fish, parvalbumin 8 (*pvalb8*) and parvalbumin 9 (*pvalb9*) belong to the oncomodulin lineage and are highly expressed in hair cells. In this study, we first reported the oncomodulin lineage function in fish and identified *pvalb8* as an essential regulator of hair cell development. Single-cell RNA sequencing (scRNA-seq) and whole-mount *in situ* hybridization (WISH) revealed that *pvalb8* is highly and specifically expressed in supporting cells and hair cells. Functional loss of *pvalb8*, achieved via CRISPR/Cas9 knockout or morpholino knockdown, resulted in reduced neuromast size and a significant decrease in neuromast hair cell number, leading to auditory behavioral deficits. In addition, *pvalb9* mutants exhibited hair cell defects similar to those observed in *pvalb8* mutants, including a significant reduction in hair cell number. Moreover, *pvalb8* loss strongly inhibited the proliferation of supporting cells, which likely accounts for the reduced number of differentiated hair cells. The expression levels of Wnt target genes, *axin2*, *ccnd1*, and *myca*, were all significantly reduced in *pvalb8* mutants compared to control zebrafish, while activation of the Wnt signaling pathway rescued the hair cell loss observed in *pvalb8* mutants, indicating that *pvalb8* promotes hair cell development via Wnt-dependent proliferative signaling. These findings highlight *pvalb8* as a critical factor in the regulation of auditory hair cell formation and function in zebrafish, offering new insights into the role of oncomodulin lineage in sensory cell development.

## 1. Introduction

According to the World Health Organization’s Global Hearing Report, approximately 20% of the population, over 1.5 billion people worldwide, experience some degree of hearing loss. This makes the development of effective treatments for deafness an urgent priority. Hearing loss arises from diverse causes, including genetic factors, aging, infectious diseases, ototoxic drugs, and noise exposure, with genetic factors being one of the leading contributors [1,2]. Gene therapy has emerged as a highly promising strategy for treating hereditary inner ear disorders [3]. In recent years, adeno-associated virus (AAV)-mediated gene delivery has demonstrated significant progress in mouse models of hearing loss, underscoring its potential for clinical translation [4,5]. Notably, the AAV-mediated delivery of a humanized OTOF gene successfully restored hearing in patients with congenital deafness caused by OTOF mutations, representing the first clinical trial of gene therapy for hereditary inner ear diseases [6,7,8]. Successful gene therapy for hereditary deafness depends on the precise identification of causative genes and an understanding of their pathogenic mechanisms. Therefore, systematic identification and functional characterization of genes associated with hearing loss could accelerate the translation of gene therapy into clinical practice.

Hair cells, as sensory receptors of the inner ear, are essential for converting external stimuli, such as sound vibrations and motion-induced accelerations, into neural signals [9]. In mammals, two distinct types of hair cells exist: outer hair cells and inner hair cells. Outer hair cells, arranged in three rows, amplify sound-induced vibrations to enhance the sensitivity of the sensory epithelium. In contrast, inner hair cells, organized in a single row, transmit these signals to spiral ganglion neurons [10]. Damage or loss of hair cells is a principal cause of hearing loss. Remarkably, in non-mammalian vertebrates such as fish, amphibians, and birds, hair cells can regenerate spontaneously after injury, enabling recovery from auditory deficits, while adult mammalian cochlear hair cells completely lose this regenerative capacity [11,12].

Calcium-binding proteins (CaBPs), including calmodulin, parvalbumin (PV), calcineurin, and S100 proteins, constitute a diverse group of proteins involved in numerous cellular processes [13]. Despite differences in their structures and properties, most CaBPs selectively and reversibly bind to Ca^2+^ through specialized domains, thereby regulating cytosolic and intracellular calcium concentrations [14]. These proteins play critical roles in various physiological systems, including the nervous and immune systems [13,14]. Recent studies have highlighted the importance of several CaBPs, such as CaBP1, CaBP2, calbindin-D28K, and S100, in the development and function of hair cells [15,16]. These reports suggest that calcium signaling is integral to the maturation and maintenance of these sensory cells, thereby implicating CaBPs as key regulators of auditory physiology. PVs are small EF-hand CaBPs characterized by two classical helix–loop–helix motifs that bind Ca^2+^ and Mg^2+^ with high affinity [17,18]. As CaBPs, they regulate intracellular calcium levels and participate in calcium-dependent signaling pathways [19]. In fish skeletal muscle, PVs are abundant and facilitate relaxation of fast-twitch fibers [20,21]. Beyond muscle, they are also expressed in the central nervous system, retina, kidney, testis, several endocrine glands, and the organ of Corti [22,23]. In the nervous system, PVs support rapid neuronal firing by enabling fast calcium clearance [24,25].

PVs were found in lower and higher vertebrates. In humans, three PV members were identified: one α-parvalbumin and two non-α isoforms, oncomodulin-1 and oncomodulin-2 (OCM1/OCM2; collectively, OCMs) [20]. Similarly, three distinct PVs have also been identified in birds: parvalbumin 1 (CPV1), parvalbumin 3 (CPV3), and Avian thymic hormone (ATH) [26]. CPV3 is homologous to the mammalian OCM and is expressed in sensory hair cells of the avian auditory organ (basilar papilla) [27]. By contrast, teleost fish express multiple PV variants with distinct tissue-specific patterns [20]. In teleost fish, the PVs fall into three lineages: α-parvalbumins (*pvalb6*, *pvalb7*), oncomodulins (*pvalb8*, *pvalb9*), and a non-α/non-oncomodulin group (*pvalb1–4*, *pvalb5*, *pvalb10*) [20]. During evolution, zebrafish did not retain the ancient *pvalb10* clade, whereas *pvalb8/9* are more conserved across species [20]. OCMs show a strikingly restricted post-embryonic expression, largely confined to subsets of inner ear sensory hair cells and, more recently, to specific immune cell types [28]. For example, OCM is a major protein in sensory cells of the guinea-pig cochlea [29]. Later studies also observed that in rats and other mammals, OCM is predominantly expressed in cochlear outer hair cells and in the striolar/central subset of vestibular hair cells [30,31]. Consistent with its expression profile, *Ocm* knockout mice develop significant progressive hearing loss [32,33]. In zebrafish, the oncomodulin lineage genes *pvalb8* and *pvalb9* are likewise highly expressed in sensory hair cells [34]. Given the calcium-regulatory function and association with hearing, investigating OCM’s specific contributions to hair cell development is essential for elucidating its mechanistic role in auditory physiology. However, despite their conserved expression profile, the roles and mechanisms of OCMs in hair cell development and hearing remain poorly defined, particularly in teleosts, highlighting a key gap addressed by our study.

The zebrafish is a powerful model organism for investigating hair cell development due to its genetic tractability, rapid embryogenesis, and optical transparency, which allow real-time visualization of developmental processes [35,36]. Therefore, zebrafish represents an efficient system for investigating the role of *pvalb8/pvalb9* in the hearing system. In this study, we demonstrate for the first time in zebrafish that oncomodulin lineage genes play a critical role in hair cell formation. Single-cell RNA sequencing (scRNA-seq) and whole-mount in situ hybridization (WISH) revealed that *pvalb8* is specifically expressed in the inner ear and neuromasts of zebrafish embryos. Functional disruption of *pvalb8* using CRISPR-Cas9 genome editing or morpholino knockdown led to significant hearing impairment and hair cell loss. Double knockout of *pvalb8* and *pvalb9* exacerbated the hair cell developmental defect. Furthermore, *pvalb8* deficiency markedly reduced cell proliferation, accounting for the observed decrease in hair cell numbers. Collectively, these findings demonstrate that *pvalb8* is essential for the development of neuromast hair cells and normal hearing function in zebrafish. Our study highlights the critical role of oncomodulin lineage genes, especially *pvalb8*, in hearing and suggests it may represent a novel deafness-associated gene, offering new insights into the molecular mechanisms underlying hair cell development and hearing loss.

## 2. Materials and Methods

### 2.1. Zebrafish Breeding

Zebrafish embryos were placed in a special culture vessel containing E3 culture medium, carefully moved into an incubator at 28 °C, and developed to 5 days post-fertilization (dpf) without feeding. At 6–15 dpf, 3 mL of Paramecium was fed twice a day with a straw. After 15 dpf, the annual worm was fed twice a day at about 11:00 and 17:00. Breeding conditions: the water temperature was maintained within the range of 26–28 °C; pH 6–7; the conductivity was between 500 and 550 μS/cm, and sodium chloride and sodium bicarbonate were regularly added to maintain pH stability.

### 2.2. Single–Cell Data Analysis

Firstly, the integrated analysis of the single-cell sequencing data, which included data filtering, data normalization, cell clustering, and cluster-level marker gene identification, was performed using Seurat V4.2.1 according to our previous study [37].

### 2.3. Whole-Mount In Situ Hybridization (WISH)

A fragment of the *pvalb8* was amplified using specific primers (Supplementary Table S1) and subcloned into the pGEM-T Easy vector. The resulting plasmid was linearized and used as a template to synthesize digoxigenin (DIG)-labeled RNA probes through in vitro transcription. WISH was performed according to protocols described in our previous work [38]. Briefly, zebrafish embryos were pretreated and hybridized overnight with the DIG-labeled probes. Following a series of stringent washes, the probes were detected using an alkaline phosphatase-conjugated anti-DIG antibody. The colorimetric reaction was developed using the BM Purple substrate to visualize the hybridization signal.

### 2.4. MO-Mediated Gene Knockdown

To inhibit the splicing of *pvalb8* pre-mRNA, a morpholino antisense oligonucleotide (*pvalb8*-MO: 5′-TCAAGTAAGACCTTCAATTAGCCAG-3′) targeting the splice junction was synthesized by Gene Tools. The lyophilized MO was dissolved in 300 µL of nuclease-free water (ddH_2_O) and stored at −20 °C. Before microinjection, 3 µL of the stock solution was diluted with 7 µL of ddH_2_O, mixed thoroughly, and heat-denatured at 65 °C for 10 min in a metal bath. To assess the efficiency of MO-mediated knockdown, reverse transcription PCR (RT-PCR) was performed using cDNA from control (ctrl) and MO-injected embryos as templates. The primers used for amplification were provided in Supplementary Table S1. PCR products were analyzed by gel electrophoresis to detect aberrant splicing or loss of target transcript, confirming MO efficacy.

### 2.5. Startle Response Experiment

The startle response assay was conducted in a quiet, dark environment. For each experimental group, approximately 50 embryos were maintained in E3 medium. At 5 dpf, 20 larvae were randomly selected and transferred into a Petri dish mounted on a mini vibrator. The acoustic stimulation parameters were configured as follows: 20 cycles with an amplitude of 9 dB re. ms^−2^, frequency of 600 Hz, pulse duration of 30 ms, and an interstimulus interval of 180 s. The swimming behavior of the larvae was recorded using an infrared camera, and the mean moving distance and peak velocity were calculated to quantify the startle response.

### 2.6. Generation of Knockout Zebrafish and Rescue Experiment

A mixture containing 300 ng/μL sgRNA and 900 ng/μL Cas9 mRNA was co-injected into zebrafish embryos at the one-cell stage. At 24 hours post-fertilization (hpf), embryos were randomly selected for genomic DNA extraction to detect indel mutations via sequencing. The primers used for genotyping mutants were provided in Supplementary Table S1. To synthesize capped mRNA for *pvalb8*, the coding sequence was amplified using gene-specific primers (Supplementary Table S1) and cloned into the pCS2+ vector. The recombinant plasmid was linearized and used as the template for in vitro transcription with the mMESSAGE mMACHINE SP6 Transcription Kit (Invitrogen, Carlsbad, CA, USA), following the manufacturer’s instructions. Purified mRNA (100–200 pg) was microinjected into one-cell stage zebrafish embryos to ensure overexpression of *pvalb8*. Rescue efficiency was calculated as $\frac{{\bar{X}}_{mRNA}-{\bar{X}}_{mut}}{{\bar{X}}_{Ctrl}-{\bar{X}}_{mut}}$ × 100%. For *pvalb8* and *pvalb9* double mutants, a mixture containing 300 ng/μL *pvalb9* sgRNA and 900 ng/μL Cas9 mRNA was co-injected into *pvalb8* mutant embryos at the one-cell stage. The genotyping method was according to *pvalb8*.

### 2.7. Quantitative RT-PCR

Total RNA was extracted from zebrafish using TRIzol reagent according to the manufacturer’s instructions (Invitrogen). Residual genomic DNA was removed with DNase I treatment. RNA quality and quantity were assessed using a NanoDrop ND-2000 spectrophotometer and agarose gel electrophoresis. Two micrograms of total RNA were reverse-transcribed into cDNA using the First Strand cDNA Synthesis Kit (Vazyme, Nanjing, China). Quantitative RT-PCR was performed with ChamQ SYBR qPCR Master Mix (Vazyme) on a 7500 Real-Time PCR System, following the manufacturer’s protocol. All samples were analyzed in three biological replicates with technical replicates. Primer sequences are listed in Supplementary Table S1.

### 2.8. Cell Proliferation and Analysis

To assess cell proliferation, zebrafish embryos at 3 dpf were incubated in E3 medium containing 10 mM 5-bromo-2′-deoxyuridine (BrdU; Sigma-Aldrich, St. Louis, MO, USA) for 24 h at 28.5 °C in 12-well plates. Then the embryos were fixed in 4% paraformaldehyde (PFA) overnight at 4 °C. These fixed larvae were rinsed three times with PBS containing 0.5% Triton X-100 (PBT-2) and then incubated in 2 N HCl at 37 °C for 30 min to denature DNA. Following acid treatment, the samples were blocked in 10% normal goat serum for 1 h at room temperature and then incubated overnight at 4 °C with a mouse monoclonal anti-BrdU antibody (1:200; Santa Cruz Biotechnology, Dallas, TX, USA). The next day, larvae were thoroughly washed with PBT-2 (three times, 10 min each), followed by incubation with a fluorescent-conjugated secondary antibody for 1 h at 37 °C. After immunostaining, larvae were imaged using a Leica TCS SP5 confocal microscope (Leica Microsystems, Wetzlar, Germany).

### 2.9. TUNEL Assay for Apoptosis Detection

To detect apoptotic signals, zebrafish embryos at 5 hpf were fixed in 4% PFA overnight at 4 °C. The fixed embryos were rinsed with PBST (PBS containing 0.1% Tween-20) for 10 min, then incubated with proteinase K for 2 min to enhance tissue permeability. After two additional PBST washes (5 min each), the embryos were re-fixed in 4% PFA for 10 min. Following re-fixation, samples were washed three times in PBST (5 min each) and subjected to TUNEL labeling using the Cell Death Detection Kit (Roche, Basel, Switzerland) in accordance with the manufacturer’s protocol.

### 2.10. FM4-64 and Wnt Activator Treatment

Wnt pathway activator, zakenpaullone (AZK) was obtained from Selleck Chemicals (Houston, TX, USA). Stock solutions were prepared by dissolving the compounds in dimethyl sulfoxide (DMSO) and subsequently diluted with E3 medium to the desired working concentrations. The zebrafish larvae were treated with a final drug concentration of 20 μM starting from 3 dpf, with observations made on 5 dpf using confocal. To visualize the MET function of lateral line neuromast hair cells, Zebrafish larvae at 5 dpf were exposed to 1 μM fluorescent vital dye FM4-64 (Invitrogen) for 15 s, followed by immediate washing 3 times with medium containing 0.17 mg/mL Tricaine (Sigma). Subsequently, the larvae were anesthetized with 0.02% MS-222, and images were taken by a confocal microscope with excitation wavelengths of 561 nm.

### 2.11. Imaging and Statistical Analysis

Following anesthesia, larvae were carefully embedded in 0.7% low-melting-point agarose in a glass-bottom dish for imaging. Confocal images were acquired and subsequently processed using Imaris software (version 649.0.1). For statistical analysis, GraphPad Prism 10 was used. Comparisons between two groups were performed using an unpaired *t*-test, while one-way ANOVA was applied for analyses involving more than two groups. A *p*-value of less than 0.05 was considered statistically significant.

## 3. Results

### 3.1. Pvalb8 Was Highly Expressed in the Neuromast Hair Cells

**Figure 1:**
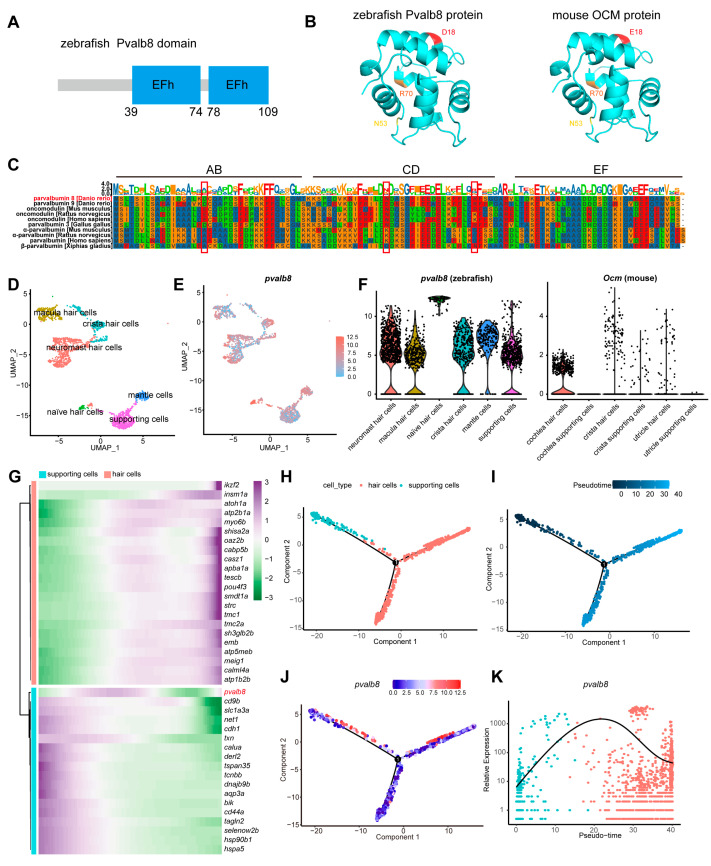
Cross-species comparison and expression pattern of *pvalb8*. (**A**) Schematic diagram showing the functional domains of the zebrafish Pvalb8 protein. (**B**) Predicted 3D structures of zebrafish Pvalb8 and mouse OCM proteins using AlphaFold, with the oncomodulin-specific residues (D/E)18, N53, and R70 highlighted. (**C**) Multiple sequence alignment of Pvalb8, Pvalb9, and homologous proteins across species, with the oncomodulin-specific residues (D/E)18, N53, and R70 highlighted by red boxes. (**D**) UMAP plot of zebrafish single-cell data showing clustering of mantle cells, supporting cells, and hair cells. (**E**,**F**) Feature plot and violin plot illustrating the expression pattern of *pvalb8*. (**G**) Heatmap showing the pseudotime expression dynamics of genes in the hair cell and supporting cell modules. (**H**,**I**) Pseudotime trajectory plots showing cell clustering and temporal dynamics. (**J**,**K**) Pseudotime trajectory and line plot displaying the dynamic expression changes in *pvalb8*.

In zebrafish, nine PVs (Pvalb1–9) have been identified. Comparative sequence and heatmap analyses showed that these proteins are highly conserved, particularly the OCM homologs Pvalb8 and Pvalb9 (Supplementary Figure S1A,B). Pvalb8 encodes a 109 amino acid protein comprising six α-helices and two EF-hand domains, in which the oncomodulin-signature residues (D/E)18, N53, and R70 are well conserved relative to mouse OCM (Figure 1A,B). Comparative sequence analysis across species revealed that Pvalb8 and Pvalb9 are highly conserved with other homologous genes, showing particularly strong homology within the EF domain (Figure 1C). Using our previously published scRNA-seq dataset of zebrafish hair cells [39], we visualized the highly *pvalb8* expression in both hair cells and supporting cells using Feature plot and violin plot, whereas the mouse ortholog *Ocm* is highly expressed in cochlear hair cells (Figure 1D–F). Moreover, violin plot analysis of all the zebrafish PVs showed that only *pvalb9* displayed an expression pattern similar to *pvalb8*, with strong enrichment in supporting cells and hair cells (Supplementary Figure S1C). Pseudotime trajectory analysis of sorted clusters further revealed that *pvalb8* expression precedes that of established hair cell markers, including *tmc1*, *myo6b*, and *pou4f3*, but follows the expression of supporting cell markers (Figure 1G). Gene expression profiling showed a significant upregulation of *pvalb8* before the differentiation of supporting cells into hair cells (Figure 1H–K). These findings indicate that *pvalb8* is dynamically regulated before hair cell formation, suggesting its potential role as an early contributor to the transition from supporting cells to mature hair cells.

**Figure 2:**
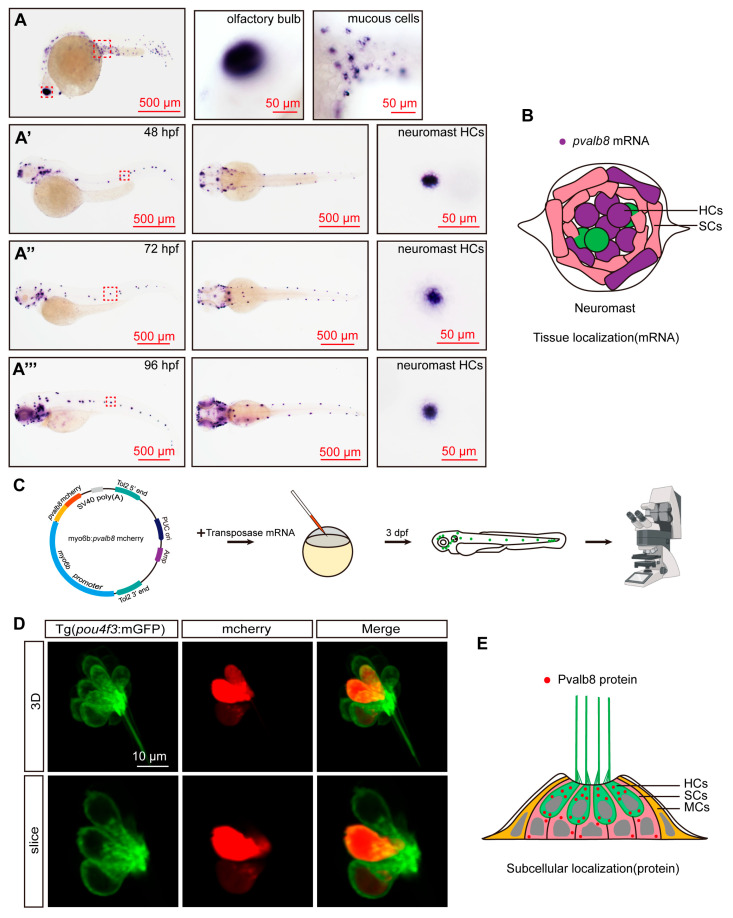
Spatiotemporal expression and subcellular localization of *pvalb8* in zebrafish. (**A**–**A**’’’) WISH results of *pvalb8* in 24, 48, 72, and 96 hpf zebrafish. The domain in the red dotted line box is magnified. (**B**) The tissue localization pattern diagram of *pvalb8* based on the combination of scRNA-seq analysis and WISH results; purple indicates the *pvalb8* mRNA expression site. (**C**) Schematic workflow of Pvalb8 fusion protein injection and confocal imaging. (**D**) The confocal imaging of hair cells labeled with *pvalb8*-mCherry fusion protein. (**E**) The subcellular localization pattern diagram of Pvalb8, based on the combination of scRNA-seq analysis and fusion protein results. The red dots indicate the Pvalb8 protein.

To further validate the expression pattern of *pvalb8* during zebrafish development, we performed WISH using an antisense probe against *pvalb8*. The results showed that during early development, *pvalb8* was highly expressed in the olfactory bulb and epidermal mucous cells (Figure 2A), consistent with previous studies [40,41]. At 48 hpf, *pvalb8* was highly expressed in the otic vesicle and neuromasts, with strong enrichment in supporting cells and hair cells, consistent with our previous scRNA-seq findings (Figure 2A’,B). As development progressed, *pvalb8* transcripts remained detectable in neuromast supporting cells and hair cells at 72 and 96 hpf (Figure 2A’’,A’’’). To investigate the subcellular localization of Pvalb8 protein, we generated a *pvalb8*-mCherry fusion construct (Figure 2C). Confocal imaging suggested that the Pvalb8 localized predominantly to the cytoplasm of hair cells (Figure 2D,E).

### 3.2. pvalb8 Is Required for Proper Neuromast Development

**Figure 3:**
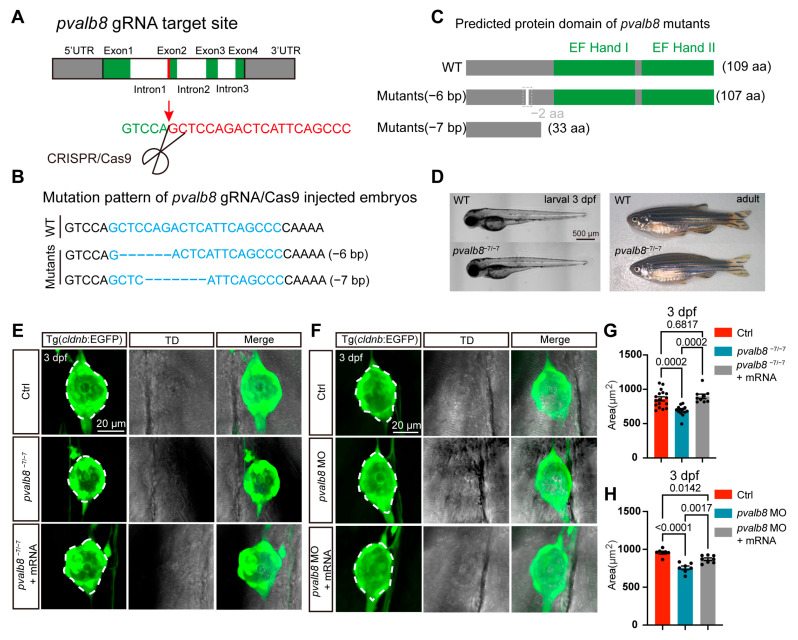
The deficiency of *pvalb8* reduced the size of the neuromast. (**A**) Schematic diagram of the *pvalb8* gene structure and the corresponding gRNA target sites. The gray box indicates the UTR region, the green box represents the exonic regions, and the white box denotes the intronic regions. The red line indicates the sgRNA target site. (**B**) Sanger sequencing analysis of the *pvalb8* mutants revealed two distinct genotypic variants: one with a 6 bp deletion and the other with a 7 bp deletion. (**C**) Schematic diagram illustrating the protein domains of the two distinct mutants. (**D**) Representative images of WT and mutant zebrafish at larval and adult stages. (**E**,**F**) Confocal images of trunk neuromasts in zebrafish at 3 dpf. The white dashed lines highlight the outer contour of the neuromast. (**G**,**H**) Quantification of the area of trunk neuromasts in zebrafish at 3 dpf (ctrl, *n* = 17, *pvalb8*^−7/−7^, *n* = 13, *pvalb8*^−7/−7^ + mRNA, *n* = 9; ctrl, *n* = 7, *pvalb8* MO, *n* = 7, *pvalb8* MO + mRNA, *n* = 9). One-way ANOVA with Tukey correction for multiple comparisons. All quantitative data are presented as Mean ± SEM.

To investigate the functional role of *pvalb8* in zebrafish, we generated a *pvalb8* knockout line using CRISPR/Cas9. The sgRNA-Cas9 system efficiently induced mutations at the targeted locus, yielding two allelic variants: a 6 bp deletion and a 7 bp deletion (Figure 3A,B). The 6 bp deletion resulted in the loss of two amino acids within a non-functional domain, while the 7 bp deletion resulted in premature translation termination and truncation of the EF-hand domain, suggesting that the mutant Pvalb8 protein loses its Ca^2+^-binding function (Figure 3C). Phenotypic examination revealed that zebrafish harboring the *pvalb8*^−7/−7^ exhibited normal larval and adult morphology compared with wild-type (WT) zebrafish (Figure 3D). We investigated the role of *pvalb8* in neuromast development by *Tg(cldnb: EGFP)* zebrafish, which express EGFP in the neuromast. Confocal imaging revealed a significant reduction in the area of trunk neuromasts in *pvalb8*^−7/−7^ mutants compared to ctrls at 3 dpf (Figure 3E,G). However, the trunk neuromast size in *pvalb8*^−6/−6^ mutants showed no differences compared with control zebrafish (Supplementary Figure S2A,B). Based on the predicted loss of function and the confocal imaging results, the *pvalb8*^−7/−7^ mutants were selected for subsequent experiments to investigate the role of *pvalb8* in neuromasts.

To further validate the specificity of the observed phenotypes and confirm the functional role of *pvalb8*, we used splice-blocking morpholino oligonucleotides (MOs) targeting *pvalb8* to downregulate *pvalb8*. Firstly, we used PCR to verify the efficiency, and the electrophoresis results showed that the *pvalb8* MO successfully induced efficient mis-splicing at the target site (Supplementary Figure S3A,B). Moreover, qRT-PCR analysis also showed that *pvalb8* expression was significantly reduced in *pvalb8* morphants compared with control zebrafish (Supplementary Figure S3C). Confocal imaging revealed that *pvalb8* morphants closely mirrored the phenotype observed in *pvalb8*^−7/−7^ mutants (Figure 3F,H). To confirm that this phenotype was specifically attributable to *pvalb8* loss-of-function, we performed a rescue experiment by injecting *pvalb8* mRNA into *pvalb8*^−7/−7^ mutants and morphant embryos. The results showed that the neuromast area was significantly restored following mRNA injection (121% for *pvalb8*^−7/−7^ mutants and 57% for *pvalb8* morphants, Figure 3E–H), supporting the specificity of the phenotype and confirming that *pvalb8* is essential for normal neuromast development.

### 3.3. pvalb8 Regulates Hair Cell Development in Neuromasts

**Figure 4:**
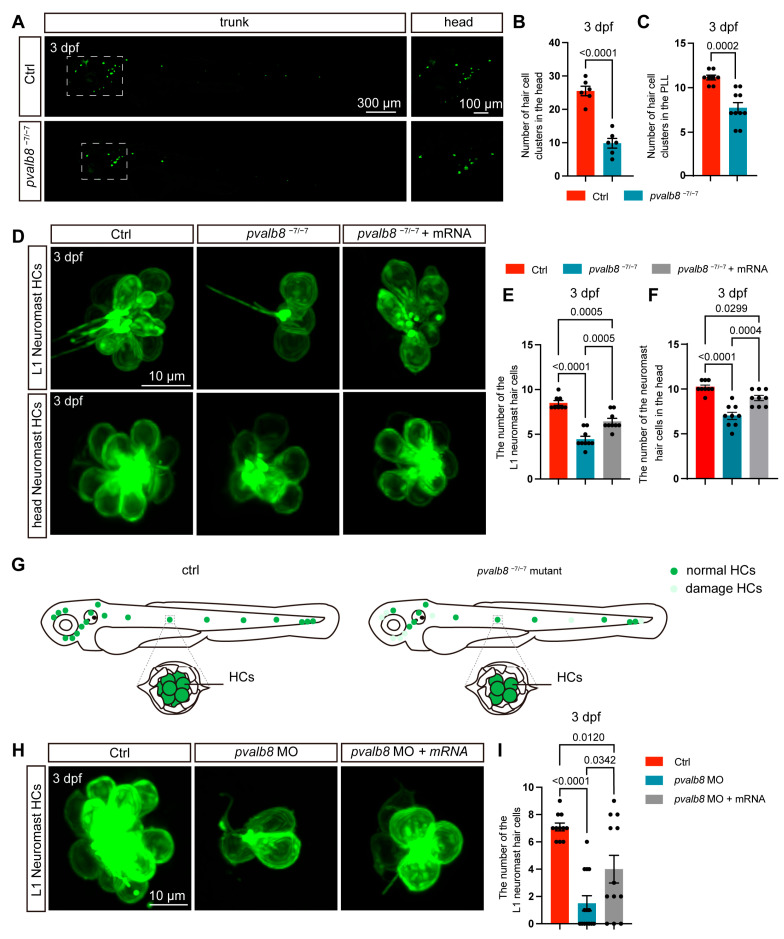
Loss of *pvalb8* reduces the number of hair cells. (**A**) Confocal images of hair cell clusters in *pvalb8^−7/−7^* and control zebrafish at 3 dpf. The magnified figure of the region squared in the dashed line is shown in the right panel. (**B**,**C**) Quantification of the hair cell cluster numbers in *pvalb8^−7/−7^* (head, *n* = 6, PLL, *n* = 10) and ctrl (head, *n* = 6, PLL, *n* = 8) zebrafish at 3 dpf. (**D**) Confocal images of hair cells in the neuromast of zebrafish L1 lateral line and head at 3 dpf across the ctrl, *pvalb8^−7/−7^*, and *pvalb8*^−7/−7^ + mRNA group. (**E**,**F**) Quantification of hair cell numbers in the ctrl (*n* = 9), *pvalb8*^−7/−7^ (*n* = 9), and *pvalb8*^−7/−7^ + mRNA group (*n* = 9). (**G**) Schematic diagram of hair cell number and distribution in the neuromasts of the ctrl and *pvalb8^−7/−7^* mutant. (**H**) Confocal images of hair cells in the L1 neuromast of zebrafish lateral line at 3 across the ctrl, *pvalb8* MO, and *pvalb8* MO + mRNA group. (**I**) Quantification of hair cell numbers in the ctrl (*n* = 11), *pvalb8* MO (*n* = 14), and *pvalb8* MO + mRNA group (*n* = 12). Student’s *t*-test for two comparisons and one-way ANOVA with Tukey correction for multiple comparisons. All quantitative data are presented as Mean ± SEM.

To assess the role of *pvalb8* in hair cell development, we examined the hair cell phenotype in *Tg(pou4f3: mEGFP)* zebrafish, whose hair cell membranes are labeled by EGFP [42]. The number of hair cell clusters and hair cells in *pvalb8*^−6/−6^ mutants was not different from that in control zebrafish (Supplementary Figure S2C–G). However, confocal imaging revealed a significant reduction in the number of hair cell clusters in the head region of *pvalb8*^−7/−7^ mutants at both 3 and 5 dpf (Figure 4A,B; Supplementary Figure S2H,I). Similarly, the formation of lateral line neuromast hair cell clusters was markedly impaired in *pvalb8*^−7/−7^ mutants (Figure 4A,C; Supplementary Figure S2H,J). To further evaluate whether *pvalb8* deficiency affects the number of hair cells within remaining clusters, we quantified the number of hair cells in both head and lateral line neuromast regions. The results showed that *pvalb8*^−7/−7^ mutants displayed a significant reduction in hair cell numbers at 3 dpf (Figure 4D–G). To further validate the specificity of the observed phenotypes, we knocked down the expression of *pvalb8* by Mo injection. The results showed that *pvalb8* morphants exhibited a marked decrease in both the number of hair cell clusters and hair cells (Figure 4H,I; Supplementary Figure S3D–F). Importantly, these defects were rescued by injection of exogenous *pvalb8* mRNA, confirming the specificity of the knockdown effect (Figure 4D–F,H,I).

Our sequence alignment and expression analyses revealed high similarity between *pvalb8* and *pvalb9*. To investigate their unique and potentially redundant roles in zebrafish hair cell development, we designed a *pvalb9* sgRNA and generated knockout zebrafish using CRISPR/Cas9 (Supplementary Figure S4A). Similar to *pvalb8*^−7/−7^ mutants, *pvalb9* mutants showed a significant reduction in neuromast hair cells compared with control zebrafish (Supplementary Figure S4B,C). Furthermore, injection of *pvalb9* sgRNA into *pvalb8*^−7/−7^ mutants resulted in more severe hair cell defects (Supplementary Figure S4D,E).

### 3.4. pvalb8 Deficiency Impairs Auditory Function

**Figure 5:**
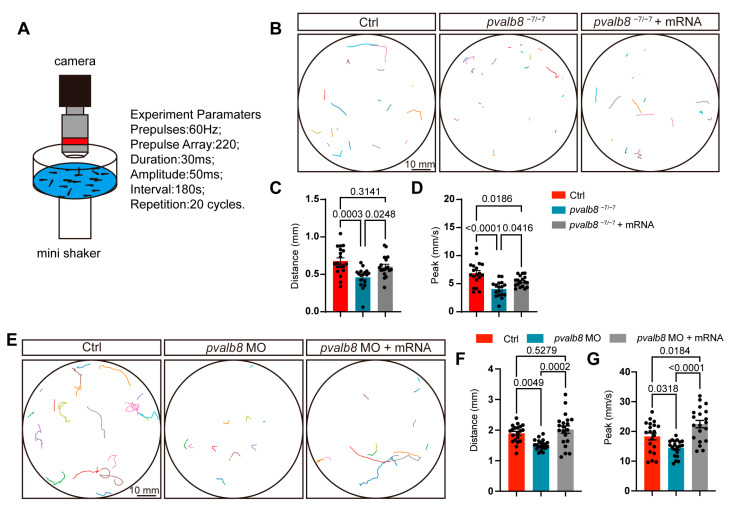
Assessment of auditory function in *pvalb8*^−7/−7^ mutants and morphants. (**A**) Schematic illustration of the startle response assay in zebrafish. (**B**) Swimming trajectories of 5 dpf zebrafish from the ctrl, *pvalb8^−7/−7^*, and *pvalb8*^−7/−7^ + mRNA group. The different colored lines represent the swimming trajectories of different fish. (**C**,**D**) Quantification of startle response distance and peak velocity (ctrl, *n* = 19, *pvalb8*^−7/−7^, *n* = 17, *pvalb8*^−7/−7^ + mRNA, *n* = 18). (**E**) Swimming trajectories of 5 dpf zebrafish from the ctrl, *pvalb8* MO, and *pvalb8* MO + mRNA group. The different colored lines represent the swimming trajectories of different fish. (**F**,**G**) Quantification of startle response distance and peak velocity (*n* = 20). One-way ANOVA with Tukey correction for multiple comparisons. All quantitative data are presented as Mean ± SEM.

We evaluated the startle response of *pvalb8*^−7/−7^ zebrafish larvae at 5 dpf to determine auditory function at *pvalb8*^−7/−7^ (Figure 5A). Using infrared video tracking, we quantified motion trajectories within 6 s post-acoustic stimulation. The results showed that control zebrafish larvae exhibited robust, immediate swimming movements, while *pvalb8*^−7/−7^ mutants displayed significantly reduced movement compared with these ctrls, indicating the remaining hair cells are insufficient to support normal auditory function (Figure 5B). Quantitative analysis revealed marked decreases in both swimming distance and peak velocity in *pvalb8*^−7/−7^ mutants (Figure 5C,D). Importantly, injection of exogenous *pvalb8* mRNA restored these deficits (65% for distance, 48% for peak), confirming the functional relevance of *pvalb8* (Figure 5B–D). Consistent results were obtained in *pvalb8* morphants, which recapitulated the mutant auditory phenotype (Figure 5E–G), indicating that *pvalb8* deficiency specifically impairs auditory function.

### 3.5. pvalb8 Deficiency Reduces Hair Cell Numbers by Impairing Proliferation Rather than Inducing Apoptosis

**Figure 6:**
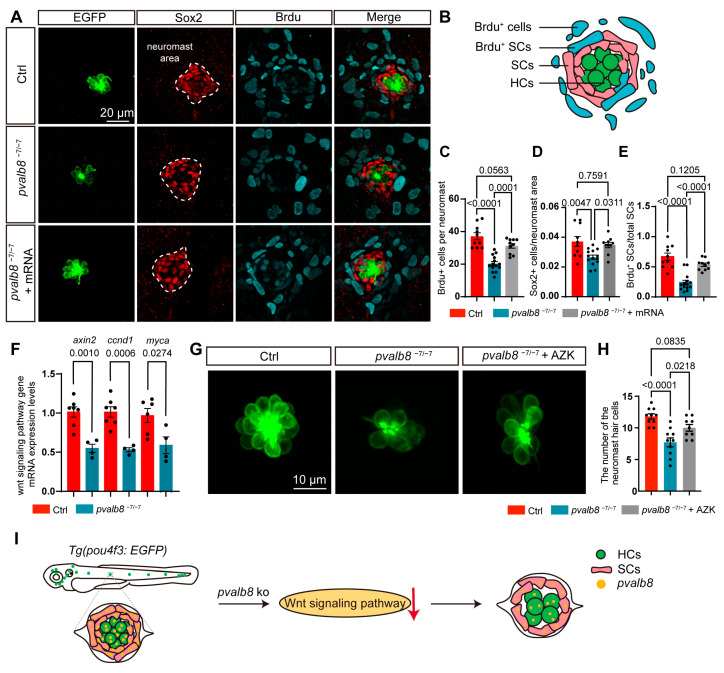
Cell proliferation and Wnt signaling activity in *pvalb8*^−7/−7^ mutants. (**A**) Confocal images of lateral neuromasts in 5 dpf ctrl, *pvalb8^−7/−7^*, and *pvalb8*^−7/−7^ + mRNA group. Hair cells were labeled with anti-GFP (green), supporting cells with anti-Sox2 (red), and proliferating cells with anti-BrdU (blue). The white dashed lines highlight the outer contour of the neuromast area. (**B**) Representative images showing hair cells, supporting cells, BrdU^+^ cells, and BrdU^+^ supporting cells in lateral line neuromasts. (**C**–**E**) Quantification of BrdU^+^ cells (ctrl, *n* = 10, *pvalb8*^−7/−7^, *n* = 14, *pvalb8*^−7/−7^ + mRNA, *n* = 10), the ratio of Sox^+^ cells to neuromast area (ctrl, *n* = 10, *pvalb8*^−7/−7^, *n* = 12, *pvalb8*^−7/−7^ + mRNA, *n* = 10), and the ratio of BrdU^+^ supporting cells to total supporting cells (ctrl, *n* = 10, *pvalb8^−7/−7^*, *n* = 14, *pvalb8*^−7/−7^ + mRNA, *n* = 10) in lateral neuromasts. (**F**) The qRT-PCR showing the reduced mRNA levels of Wnt target genes in the *pvalb8*^−7/−7^ mutants (*n* = 4) compared to control zebrafish (*axin2*, *n* = 7, *ccnd1*, *n* = 7, *myca*, *n* = 6). Student’s *t*-test. (**G**) Confocal images of hair cells in lateral line neuromasts at 5 dpf across the ctrl, *pvalb8^−7/−7^*, and AZK-treated group. (**H**) Quantification of hair cell numbers in lateral line neuromasts (ctrl, *n* = 10, *pvalb8*^−7/−7^, *n* = 10, *pvalb8*^−7/−7^ + AZK, *n* = 9). (**I**) The schematic diagram illustrates the progressive reduction of hair cells and supporting cells in the zebrafish neuromast following *pvalb8* knockout. The red arrow indicates the downregulation of the Wnt signaling pathway. One-way ANOVA with Tukey correction for multiple comparisons. All quantitative data are presented as Mean ± SEM.

Given that auditory hair cells primarily rely on mechanotransduction (MET) channels, to assess whether the hearing loss in *pvalb8*^−7/−7^ mutants was associated with functional defects of hair cells, we used FM4-64, a styrene dye that rapidly enters hair cells through MET channels, to evaluate hair cell activity [43]. Confocal imaging and quantitative analysis showed that FM4-64 fluorescence intensity in *pvalb8*^−7/−7^ mutants was not significantly reduced compared to ctrls, indicating that the remaining hair cells in *pvalb8*^−7/−7^ mutants retain normal MET channel function (Supplementary Figure S5A,B). Given the significant reduction in hair cell number, we investigated whether this phenotype resulted from increased apoptosis or impaired proliferation. However, TUNEL staining revealed no apoptotic signals in hair cells of *pvalb8*^−7/−7^ mutants (Supplementary Figure S5C,D), excluding apoptosis as the primary cause. To further examine the impact of *pvalb8* on cell proliferation, we conducted BrdU incorporation assays by treating larvae with 10 mM BrdU from 3 to 5 dpf, followed by immunofluorescence labeling. We first quantified BrdU^+^ cells in lateral line neuromasts. Control larvae showed an average of 37.10 ± 2.2 BrdU^+^ cells per neuromast, while *pvalb8*^−7/−7^ mutants exhibited a significant reduction, which was partially restored upon *pvalb8* mRNA injection (78%, Figure 6A–C). Given that supporting cells are the main progenitors for hair cell regeneration, we specifically examined their proliferation. The percentage of Sox2^+^ supporting cells/neuromast area and the percentage of Sox2/BrdU double-positive cells were significantly lower in *pvalb8*^−7/−7^ mutants compared to ctrls (Figure 6D,E), indicating impaired proliferative capacity within this progenitor pool.

Wnt signaling regulates supporting cell proliferation and influences hair cell development and regeneration [44]. We want to know whether the Wnt signaling was affected by the deficiency of *pvalb8*. Therefore, we examined the expression of Wnt target genes, *axin2*, *ccnd1*, and *myca*, in *pvalb8^−7^/^−7^* mutants and control zebrafish using qRT-PCR. The results showed that expression of *axin2*, *ccnd1*, and *myca* was significantly reduced in the *pvalb8*^−7/−7^ mutants compared with ctrls (Figure 6F), indicating that *pvalb8* is associated with Wnt/β-catenin activity. Therefore, to further test whether activating Wnt signaling could rescue the hair cell defects in *pvalb8*^−7/−7^ mutants, we further treated zebrafish larvae with azakenpaullone (AZK), a Wnt pathway activator, from 3 to 5 dpf. The results revealed that AZK treatment significantly increased the number of hair cells (56%) in *pvalb8*^−7/−7^ mutants compared to untreated mutants (Figure 6G,H), indicating that pharmacological activation of Wnt signaling can partially rescue the hair cell deficits caused by *pvalb8* deficiency (Figure 6I).

## 4. Discussion

In mammals, hearing loss primarily results from the irreversible degeneration of inner ear hair cells [45]. Thus, identifying genes involved in hair cell development and elucidating their underlying mechanisms are critical for advancing the prevention and treatment of hereditary hearing. CaBPs perform diverse cellular functions, including the regulation of cell growth and differentiation, membrane excitability, and neurotransmitter release [46]. Despite the recognized importance of calcium signaling in sensory systems, the specific role of CaBPs in auditory hair cells remains poorly understood. In this study, we identified the oncomodulin lineage gene, *pvalb8*, as a CaBP highly expressed in zebrafish hair cells and demonstrated its essential role in hair cell formation and function.

Our scRNA-seq analysis and WISH revealed that *pvalb8* is highly expressed in supporting cells and hair cells, suggesting a critical role in hair cell development or function. Subcellular localization studies demonstrated that Pvalb8 is predominantly cytoplasmic within hair cells, consistent with previous findings where adenoviral expression of parvalbumin–DsRed fusion protein (PV-DsRed) localized PV to the cytoplasm in vitro [47]. It has been reported that Pvalb8 is orthologous to mammalian OCM, likely arising from the teleost-specific genome duplication event [48]. In this study, comparative sequence and predicted 3D structures analysis across species revealed that Pvalb8 is highly conserved with OCM in mice. Although nine PV variants are present in zebrafish, we found that only *pvalb9*, another oncomodulin lineage gene, shares the highest similarity sequence and expression profile with *pvalb8*, with strong enrichment in supporting cells and hair cells. Similarly, comparison of the top 200 protein-coding gene orthologs expressed in zebrafish hair cells and mouse inner hair cells confirmed that zebrafish *pvalb8* and *pvalb9*, together with mouse *Ocm*, are highly expressed in hair cells [34]. These findings support that zebrafish *pvalb8* and *pvalb9* are orthologous to mammalian *Ocm*, which might be essential for hair cell development and hearing function. Moreover, the two oncomodulin lineage genes, *pvalb8* and *pvalb9*, may play redundant or compensatory roles due to their sequence similarity and overlapping expression patterns.

In zebrafish, lateral line neuromasts are composed of hair cells surrounded by supporting cells, which are encircled by an outer ring of mantle cells [49]. Live imaging of neuromast-specific transgenic zebrafish, coupled with the species’ genetic tractability, has provided valuable insights into sensory organ development [50]. In this study, we observed a significant reduction in the number of neuromast hair cells in *pvalb8* mutants and morphants at an early stage, although many hair cell clusters remained intact. Moreover, loss of *pvalb9* also led to a reduction in hair cells. The phenotypic similarities between *pvalb8* and *pvalb9* mutants, together with their co-expression, suggest that the two genes function within the same pathway and cooperatively regulate hair cell development. Moreover, the more severe defects of hair cells in the double knockout of *pvalb8* and *pvalb9* further indicate functional redundancy between the two genes in the developing auditory system. This redundancy may also explain the residual hair cells observed in *pvalb8* mutants with compromised *pvalb9* function.

Functionally, lateral line neuromasts are analogous to the mechanosensory receptors of the mammalian cochlea. Hair cells within these neuromasts detect acoustic stimuli and local water movement or pressure changes, converting mechanical signals into chemical and electrical cues that activate auditory pathways [51]. In this study, startle response assays revealed marked reductions in swimming distance and speed after stimuli in *pvalb8* mutants at 5 dpf compared to ctrls, indicating that remaining hair cells were insufficient to sustain normal auditory function. Notably, the MET currents of the remaining hair cells were preserved, arguing that the deficit stems primarily from insufficient hair cell number, rather than an intrinsic mechanotransduction defect in surviving cells. In mice, loss of *Ocm* causes progressive hearing loss with elevated auditory brainstem response (ABR) thresholds and reduced distortion product otoacoustic emission (DPOAEs) at 14–26 weeks, but sound-evoked responses remain normal at 4 weeks [32,33], which is different from the early-stage defects in zebrafish losing *pvalb8*. This phenotype difference in temporal profile after *Ocm* deficiency between zebrafish and mice likely reflects species-specific developmental timelines. In mice, many aspects of cochlear hair cell maturation occur postnatally, whereas in zebrafish, inner ear hair cells differentiate and function during embryonic and early larval stages [52,53]. Moreover, zebrafish oncomodulin lineage genes (*pvalb8* and *pvalb9*) are not identical to mouse *Ocm*, which may lead to partial functional differences. However, whether *pvalb8* also plays a role in maintaining hair cell survival and auditory function, like mouse *Ocm*, warrants further investigation.

Organ size is determined largely by the number and size of its constituent cells, which are regulated by cell growth, apoptosis, and proliferation [54]. In this study, the trunk neuromast size of *pvalb8* mutants was reduced, accompanied by a significant decrease in both supporting cell and hair cell numbers. We observed no apoptotic signals in *pvalb8* mutant neuromasts, but BrdU labeling revealed a marked reduction in proliferating supporting cells. Supporting cells are known to reenter the cell cycle, proliferate, and subsequently differentiate into new hair cells, thereby contributing to hair cell formation [9]. These findings suggest that impaired supporting cells proliferation in *pvalb8* mutants might underlie the reduction in neuromast size and hair cell number. The Wnt signaling pathway is a well-established regulator of developmental processes, including cell proliferation, fate determination, and tissue patterning [55,56]. In this study, the deficiency of *pvalb8* significantly suppresses the expression of Wnt target genes and activation of Wnt signaling, with azakenpaullone (AZK) significantly rescuing the number of neuromast hair cells in *pvalb8* mutants. These results suggest that *pvalb8* may regulate hair cell formation, at least in part, by modulating Wnt-dependent proliferative mechanisms.

Gene therapy offers significant promise for treating hereditary hearing loss. Recent advances in AAV-mediated gene delivery have produced encouraging results in mouse models of deafness, underscoring the feasibility of this approach for auditory disorders [7]. A critical prerequisite, however, is the identification of disease-causing genes and the mechanisms through which they act. Although PV members have not previously been associated with human deafness, OCM is essential for cochlear hair cells, where it regulates spontaneous calcium signaling and supports maturation of afferent innervation [57]. In this study, we show that *pvalb8* contributes to hair cell development and auditory function in zebrafish. Given their strong genetic homology with mammals, zebrafish auditory hair cells share structural, functional, and molecular features with mammalian inner ear hair cells [35,58]. Together, these findings suggest that OCM may represent a potential deafness gene. With large-scale population screening and detailed functional studies, OCM could emerge as a molecular marker for hearing and balance disorders, as well as a viable therapeutic target.

## 5. Conclusions

In conclusion, our study demonstrates that *pvalb8* is indispensable for the development and function of neuromast hair cells in zebrafish. By clarifying its role in supporting cell proliferation and hair cell formation, we provide new insights into the regulatory mechanisms of OCM in auditory hair cell development and maintenance. These findings not only advance our understanding of hair cell biology but also highlight OCM as a potential target for future strategies to prevent or treat hearing disorders. Nonetheless, several limitations should be acknowledged. The zebrafish auditory system is not identical to that of mammals. Electrophysiological measurements, such as calcium imaging in hair cells or auditory evoked potentials, which directly assess hair cell function, were not performed. In addition, the mechanistic links between *pvalb8*, Wnt signaling, and *pvalb9* remain unclear. Addressing these gaps will require further studies to validate and extend our findings.

## Data Availability

The original contributions presented in this study are included in the article/Supplementary Materials. Further inquiries can be directed to the corresponding authors.

## Supplementary Materials

The following supporting information can be downloaded at: https://www.mdpi.com/article/10.3390/cells14191572/s1, Figure S1: Expression abundance and protein sequence similarity of the zebrafish parvalbumin family. Figure S2: Deletion of Pvalb8 functional domains reduces the number of hair cells. Figure S3: *pvalb8* knockdown reduces the number of hair cell clusters. Figure S4: *pvalb9* deficiency caused a reduction in the number of hair cells. Figure S5: Assessment of hair cell functionality and apoptosis in *pvalb8*^−7/−7^ mutants. Table S1: Summary of primers and gRNAs.
