# Supplementary material for: Pvalb8, a Type of Oncomodulin, Regulates Neuromast Development and Auditory Function in Zebrafish
Source: Cells. 2025 Oct 9;14(19):1572. doi: 10.3390/cells14191572 (PMC12523338; doi:10.3390/cells14191572)
Supplement: Supplementary file 1 [file cells-14-01572-s001.zip › cells-3857010-supplementary.pdf]

Pvalb8, a Type of Oncomodulin, Regulates Neuromast Development and Auditory Function in Zebrafish

Guiyi Zhang[1,†], Qianqian Li[1,†], Ying Xu[1], Hanmeng Zhao[1], Chao Yang[2], Dong Liu[1,2*], Jie Gong[1*]

1 School of Life Sciences, Nantong University, Nantong 226001, China; 2309310004@stmail.ntu.edu.cn (G.Z.); lqianqian2022@126.com (Q.L.); 2409310002@stmail.ntu.edu.cn (Y.X.); 2409110106@stmail.ntu.edu.cn (H.Z.)

2 Key Laboratory of Neuroregeneration of Jiangsu and Ministry of Education, Co-innovation Center of Neuroregeneration, Nantong University, Nantong, China; 2225510003@stmail.ntu.edu.cn (C.Y)

* Correspondence: tom@ntu.edu.cn (D.L.); jgong188@ntu.deu.cn (J.G.); Tel.: +(86)-18605133927 (D.L)

† These authors contributed equally to this work.

Contact details for Correspondence:

Dong Liu, Ph.D; Jie Gong, Ph.D

Nantong Laboratory of Development and Diseases, Nantong University

Seyuan Road 9, Nantong, China, 226001

Phone: + (86) - 18605133927

Email: liudongtom@gmail.com; jgong188@ntu.deu.cn

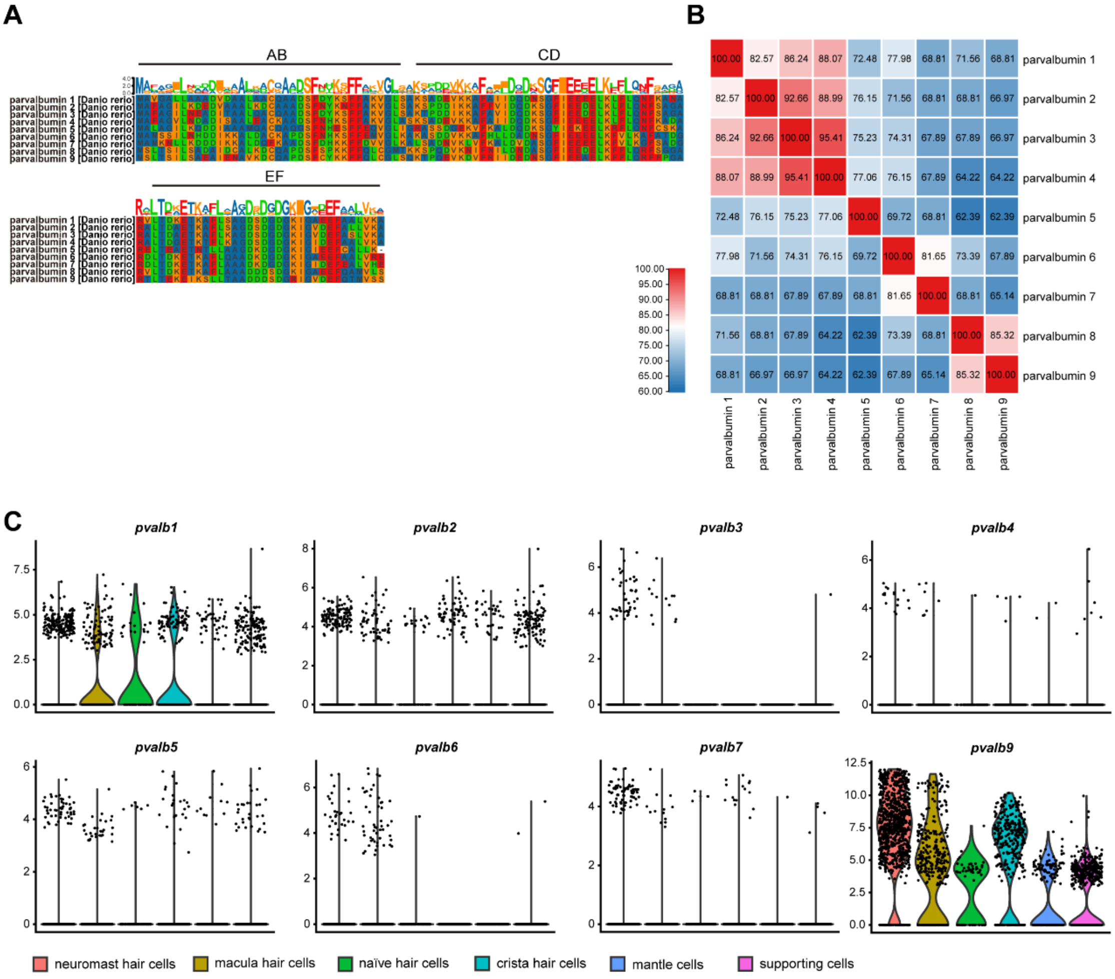

Supplementary Figure 1. Expression abundance and protein sequence similarity of the zebrafish parvalbumin family. (A) Violin plots illustrating the expression pattern of *pvalb1*, *2*, *3*, *4*, *5*, *6*, *7*, and *9*. (B) Multiple sequence alignment of 9 Parvalbumins in zebrafish. (C) Heatmap showing sequence similarity among 9 parvalbumins.

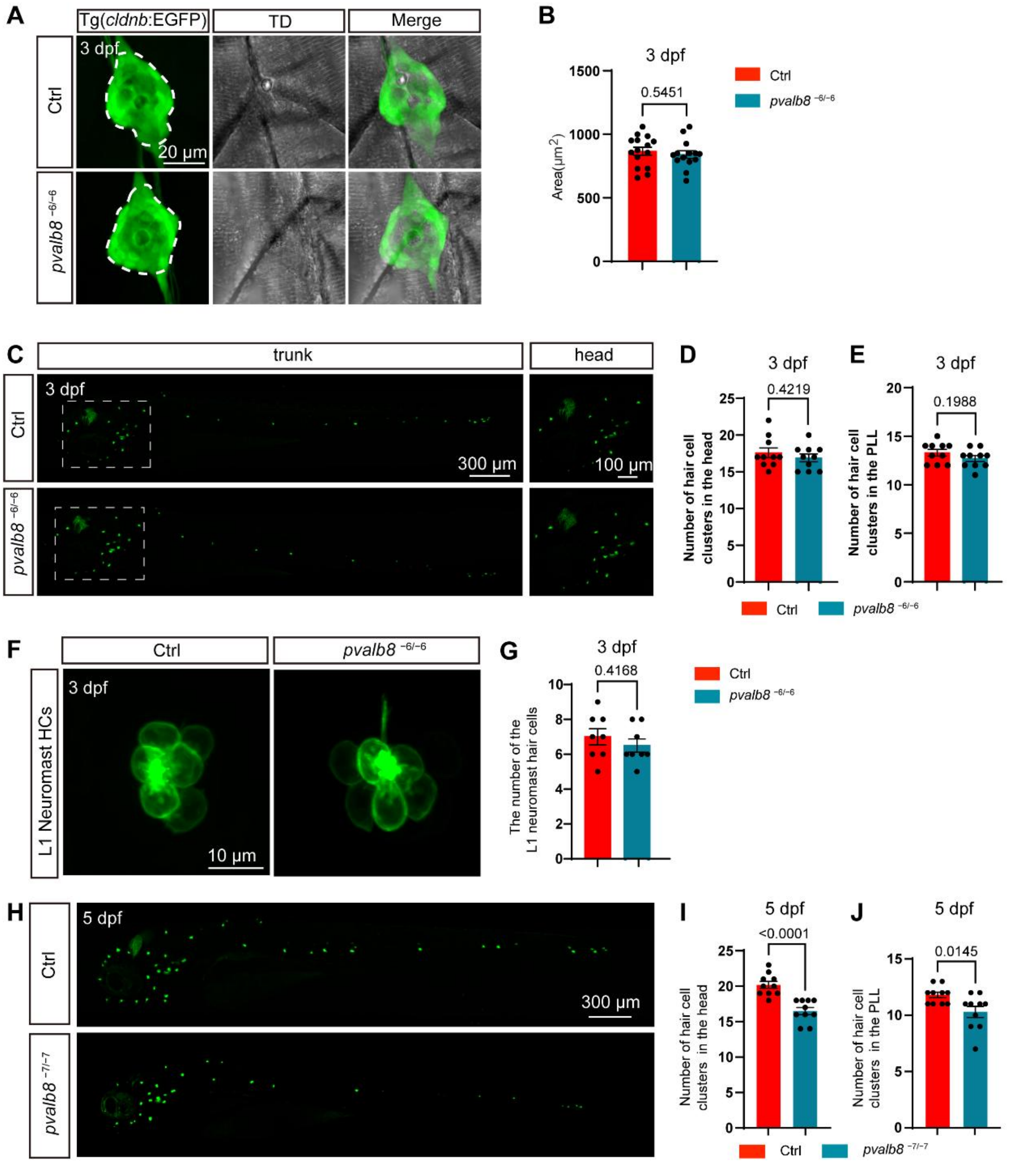

Supplementary Figure 2. Deletion of Pvalb8 functional domains reduces the number of hair cells. (A) Confocal images of trunk neuromasts in *pvalb8*$^{-6/-6}$ mutants at 3 dpf. The white dashed lines highlight the outer contour of the neuromast. (B) Quantification of the area of trunk neuromasts in *pvalb8*$^{-6/-6}$ (n = 14) and ctrl (n = 15) at 3 dpf. (C) Confocal images of hair cell clusters in *pvalb8*$^{-6/-6}$ and ctrl at 3 dpf. The magnified figure of the region squared in the dashed line is shown in the right panel. (D, E) Quantification of the hair cell cluster numbers in *pvalb8*$^{-6/-6}$ (head, n = 10, PLL, n = 10) and ctrl (head, n = 10, PLL, n = 10) at 3 dpf. (F) Confocal images of hair cells in the neuromast of zebrafish L1 lateral line in *pvalb8*$^{-6/-6}$ and ctrl at 3 dpf. (G) Quantification of hair cell numbers in *pvalb8*$^{-6/-6}$ (n = 8) and ctrl (n = 8) at 3 dpf. (H) Confocal images of hair cell clusters in *pvalb8*$^{-7/-7}$ and ctrl at 5 dpf. (I, J) Quantification of the hair

cell cluster numbers in *pvalb8*$^{-7/-7}$ (head, n = 10, PLL, n = 10) and ctrl (head, n = 10, PLL, n = 10) at 5 dpf. Student's t-test for two comparisons. All quantitative data are presented as Mean ± SEM.

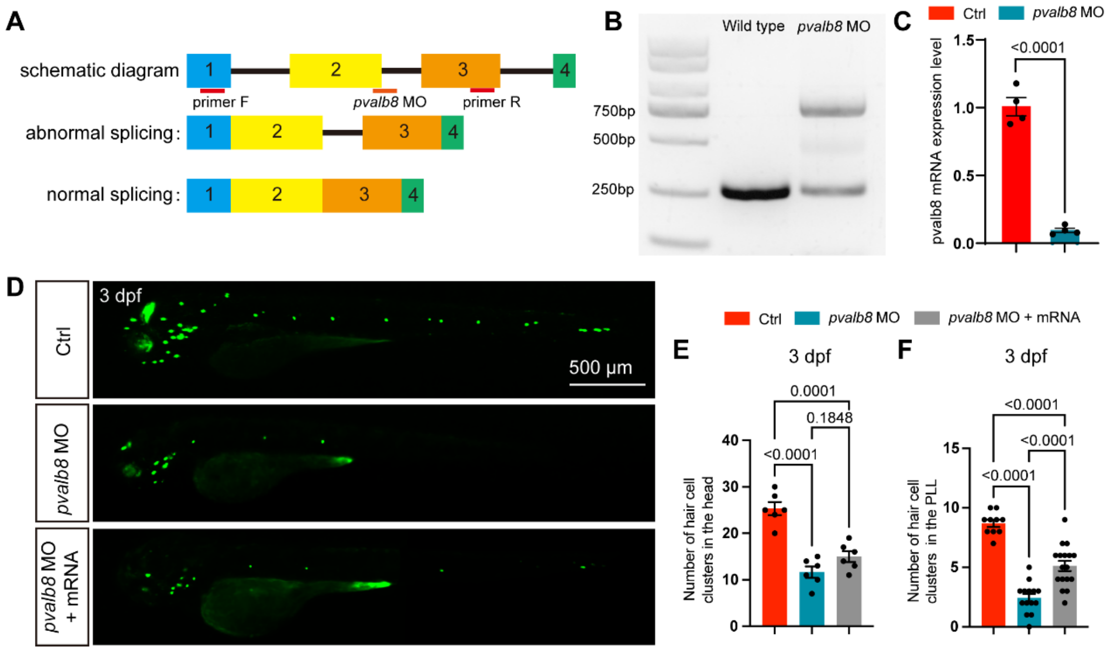

Supplementary Figure 3. *pvalb8* knockdown reduces the number of hair cell clusters. (A) Schematic diagram of *pvalb8* MO design and its splicing interference mechanism. (B) Agarose gel electrophoresis to evaluate splice-blocking efficiency of *pvalb8* MO. (C) The qRT-PCR showing the reduced mRNA level of *pvalb8* in the *pvalb8* MO (n = 4) compared to ctrl (n = 4). (D) Confocal images of hair cell clusters in the ctrl, *pvalb8* MO, and *pvalb8* MO + mRNA group at 3 dpf. (E, F) Quantification of the hair cell cluster numbers in the ctrl (head, n = 6, PLL, n = 10), *pvalb8* MO (head, n = 6, PLL, n = 14), and *pvalb8* MO + mRNA group (head, n = 6, PLL, n = 17). Student's t-test for two comparisons and one-way ANOVA with Tukey correction for multiple comparisons. All quantitative data are presented as Mean ± SEM.

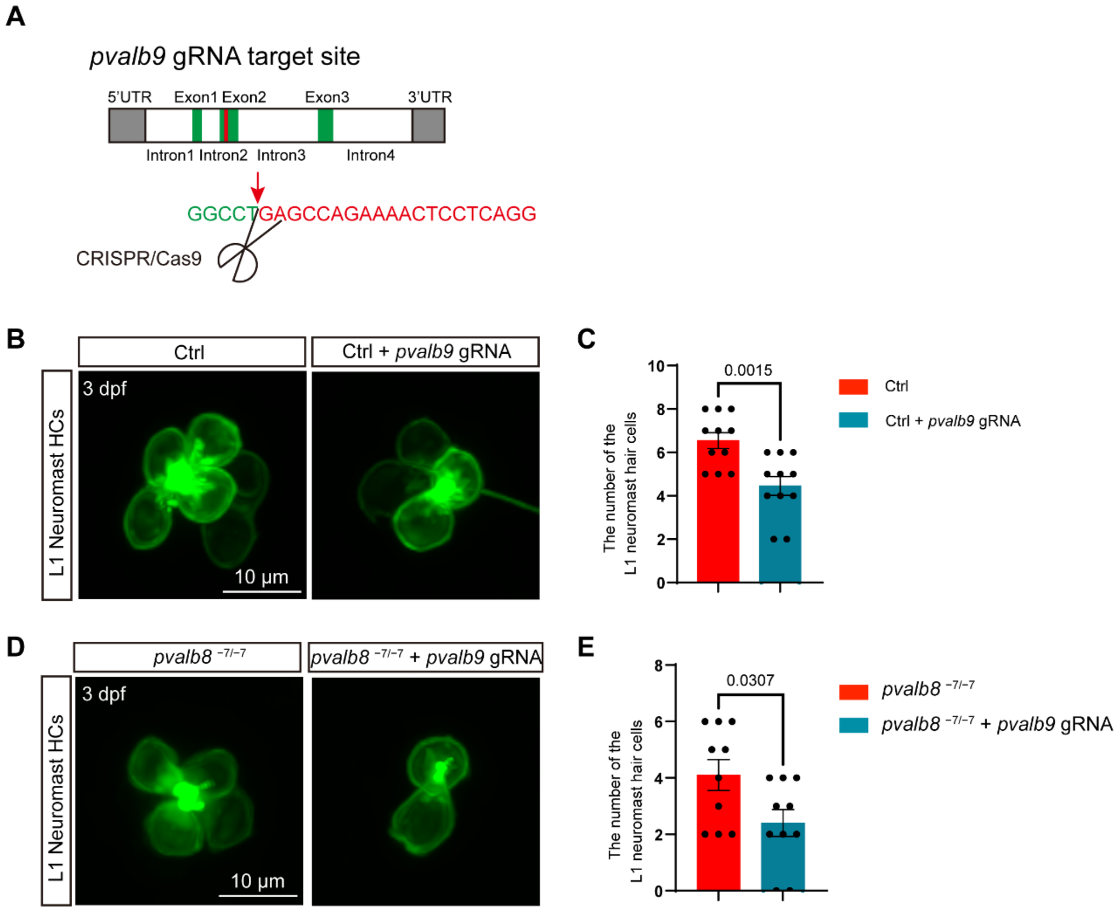

Supplementary Figure 4. *pvalb9* deficiency caused a reduction in the number of hair cells. (A) Schematic diagram of the *pvalb9* gene structure and the corresponding sgRNA target sites. The gray box indicates the UTR region, the green box represents the exonic regions, and the white box denotes the intronic regions. The red text indicates the sgRNA target site. (B) Confocal images of hair cells in the neuromast of zebrafish L1 lateral line in *pvalb9* sgRNA injection and ctrl group at 3 dpf. (C) Quantification of hair cell numbers in *pvalb9* sgRNA injection (n = 11) and ctrl group (n = 11) at 3 dpf. (D) Confocal images of hair cells in the neuromast of zebrafish L1 lateral line in *pvalb8*$^{-7/-7}$ injected with *pvalb9* sgRNA and *pvalb8*$^{-7/-7}$ at 3 dpf. (E) Quantification of hair cell numbers in *pvalb8*$^{-7/-7}$ injected with *pvalb9* sgRNA (n = 10) and *pvalb8*$^{-7/-7}$ (n = 10) zebrafish at 3 dpf. Student's t-test for two comparisons. All quantitative data are presented as Mean ± SEM.

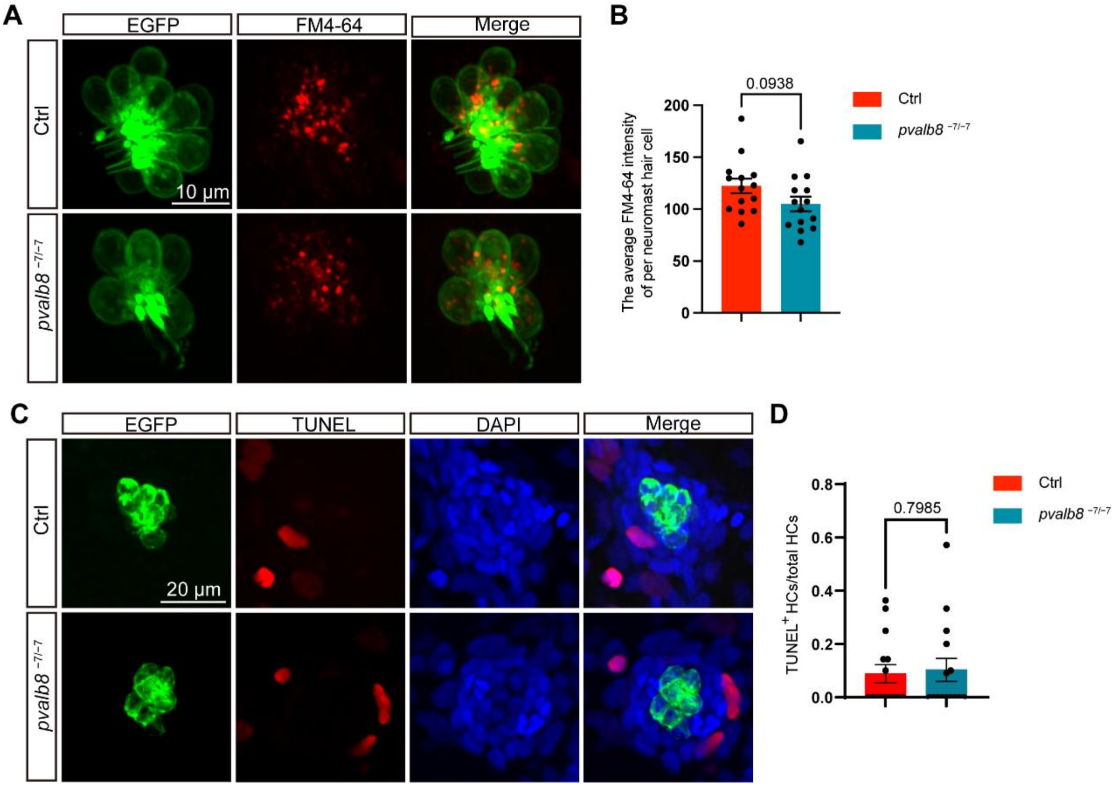

Supplementary Figure 5. Assessment of hair cell functionality and apoptosis in *pvalb8*$^{-7/-7}$ mutants. (A) Representative confocal images of lateral neuromast hair cells from *pvalb8*$^{-7/-}$ and ctrl at 5 dpf following FM4-64 uptake. Hair cells are labeled with green fluorescence, and those that successfully incorporated FM4-64 exhibit red fluorescence. (B) Quantification of the average FM4-64 fluorescence intensity per group (ctrl, n = 14, *pvalb8*$^{-7/-7}$, n = 14). (C) Representative confocal images of TUNEL apoptosis assay in *pvalb8*$^{-7/-7}$ and ctrl. Hair cells are labeled with green fluorescence, TUNEL^{+} apoptotic signals are shown in red, and nuclei are stained blue. (D) Quantification of the ratio of the TUNEL^{+} HCs to total HCs (ctrl, n = 14, *pvalb8*$^{-7/-7}$, n = 15). Student's t-test for two comparisons. All quantitative data are presented as Mean ± SEM.

Supplementary Table 1. Summary of primers and gRNAs

Primers	Primer Sequence (5'- 3')	Purpose
pvalb8-MO-F	TCGACTGTGCACTCAAGGAC	used for RT-PCR
pvalb8-MO-R	CAATCTTGCCATCGCTGTCG	used for RT-PCR
pvalb8-mut-F	CAGCTATTGTAGTCGAAGACAA	used for genotyping
pvalb8-mut-R	GAAACCCACGCTAACATGG	used for genotyping
pvalb8-mRNA-F	CGCGGATCCCTGTTCATTCACCTGCCGGA	used for overexpression
pvalb8-mRNA-R	CCGGAATTCAAAACACCGGCGTATCGAGT	used for overexpression
pvalb8-probe-F	ATGTCTCTCACATCTATCCT	used for *in situ* hybrdization
pvalb8-probe-R	TCAGGACAGCACCATCGCCT	used for *in situ* hybrdization
pvalb9-mut-F	AGACTGCCAAGGTGTGGTAA	used for genotyping
pvalb9-mut-R	GCATTAAGAAGTGCCTCCTCTG	used for genotyping
q-*pvalb8*-F	GCCCTCAGGATGTGAAGAACA	used for qRT-PCR
q-*pvalb8*-R	GCCTGGAACTCTTCTGCTCC	used for qRT-PCR
q-*ccnd1*-F	TTGCAAATGGAACTGCTGGC	used for qRT-PCR
q-*ccnd1*-R	AGGGCTTGCGATGAAGTTGA	used for qRT-PCR
q-*myca*-F	CCTCGCACATCGGATTCTGA	used for qRT-PCR
q-*myca*-R	TGCACTCTGTCGCCTTCTTT	used for qRT-PCR
q-*axin2*-F	TCTAGTGATGCCCTGACGGA	used for qRT-PCR
q-*axin2*-R	GTCATCTCCTTAGGTGGCCG	used for qRT-PCR
pvalb8-gRNA	GGGCTGAATGAGTCTGGAGC	gRNA sequence
pvalb9-gRNA	GGGCCAGAAAACTCCTCAGG	gRNA sequence
